# Microbiota-Derived L-SeMet Potentiates CD8^+^ T Cell Effector Functions and Facilitates Anti-Tumor Responses

**DOI:** 10.3390/ijms26062511

**Published:** 2025-03-11

**Authors:** Simiao Fan, Yaxin Li, Shaoyi Huang, Wen Wang, Biyu Zhang, Jiamei Zhang, Xiaoxiao Jian, Zengqing Song, Min Wu, Haiqing Tu, Yuqi Wen, Huiyan Li, Sen Li, Huaibin Hu

**Affiliations:** 1 Nanhu Laboratory, National Center of Biomedical Analysis, Beijing 100850, China; fan_simiao@163.com (S.F.); lyxyd2019@163.com (Y.L.); syhuang@nanhulab.ac.cn (S.H.); wwang@nanhulab.ac.cn (W.W.); byzhang@xmail.ncba.ac.cn (B.Z.); xxjian@xmail.ncba.ac.cn (X.J.); zqsong@xmail.ncba.ac.cn (Z.S.); mwu@xmail.ncba.ac.cn (M.W.); hqtu@xmail.ncba.ac.cn (H.T.); wenyuqi7@163.com (Y.W.); hyli@ncba.ac.cn (H.L.); 2 School of Basic Medical Sciences, Fudan University, Shanghai 200032, China; 22111010092@m.fudan.edu.cn

**Keywords:** microbial metabolites, L-selenomethionine, CD8^+^ T cell, anti-tumor therapy

## Abstract

Extensive studies have shown that gut microbiota-derived metabolites can enhance the antitumor efficacy of immunotherapy by modulating host immune responses. However, the more comprehensive spectrum of such metabolites and their mechanisms remain unclear. In this study, we demonstrated that L-selenomethionine (L-SeMet), a gut microbial metabolite, acts as a positive regulator of immunotherapy. Through screening of a repository of gut microbial metabolites, we identified that L-SeMet can effectively enhance the effector function of CD8^+^ T cells. Furthermore, intragastric administration of L-SeMet in mice significantly suppressed the growth of subcutaneous MC38 tumors. Mechanistically, L-SeMet enhances T cell receptor (TCR) signaling by promoting LCK phosphorylation. Collectively, our findings reveal that the gut microbial metabolite L-SeMet inhibits colorectal tumor growth by potentiating CD8^+^ T cell functions, providing a potential therapeutic strategy for colorectal cancer treatment.

## 1. Introduction

Tumor immunotherapy has exhibited sustained and effective clinical responses [1,2]. Tumor-reactive CD8^+^ T cells play a pivotal role in mediating potent cytotoxic immune responses. However, within the tumor microenvironment (TME), the functional impairment of CD8^+^ T cells often suppresses anti-tumor immunity, leading to poor clinical outcomes in many cancer therapies [3,4]. Consequently, strategies to enhance the functionality of CD8^+^ T cells within the TME are of significant interest for improving cancer treatment efficacy.

Emerging evidence highlights the role of the gut microbiota in modulating the host immune system and influencing the therapeutic efficacy of immunotherapy [5,6,7,8]. Although immune checkpoint inhibitors, including PD-1/PD-L1 and CTLA-4 antibodies, have become essential tools in cancer treatment, their clinical response rates remain relatively unsatisfactory. Importantly, *Clostridials*, *Faecalibacterium*, or *Ruminococcaceae* ameliorate the antitumor efficacy of ICI in melanoma patients [9]. For instance, oral administration of *Bifidobacterium longum* in mice demonstrated improved ICI therapy efficacy through enhancing interferon-gamma (IFN-γ) production in CD8^+^ T cells [10]. Moreover, a mixture of 11 bacterial strains isolated from healthy human feces has been shown to strengthen the anti-tumor efficiency of PD-1 blockade combination therapy [11]. Furthermore, there is a lack of reliable biomarkers to predict patient responses to immunotherapy in clinical practice. Specific gut microbiota compositions have been identified as potential predictors of ICI treatment outcomes [9,10,12,13]. By analyzing microbiome profiles in fecal or blood samples, predictive models can be developed to identify patients likely to respond to immunotherapy, thereby improving the success rate of personalized treatment strategies. In addition to modulating ICI immunotherapy, gut microbiota can also influence the efficiency of chimeric antigen receptor (CAR) T cell therapy [14] and adoptive T cell transfer (ACT) immunotherapy [15]. Collectively, these crucial roles of gut microbiota in cancer immunotherapy will provide new directions for future cancer treatment.

The gut microbiota produces numerous metabolites, some of which have been demonstrated to modify tumor progression and immune responses [16,17]. For example, the secondary bile acid deoxycholic acid (DCA) has been shown to suppress CD8^+^ T cell effector function and promote tumor growth [18]. The tryptophan-derived metabolite indole-3-acetic acid (3-IAA) has been found to potentiate the effectiveness of chemotherapy in pancreatic ductal adenocarcinoma (PDAC) [19]. Short-chain fatty acids (SCFAs), such as valerate and butyrate, boost the anti-tumor activity of cytotoxic T lymphocytes (CTLs) and CAR-T cells by modifying metabolic pathways and epigenetic reprogramming [20]. Notably, butyrate also promotes the memory potential of CD8^+^ T cells [21]. Collectively, these findings indicate that modulating gut microbiota metabolites may represent a promising therapeutic strategy for cancer treatment.

Selenium (Se) is a vital trace element for mammals, naturally present in soil, water, and various foods. The biological functions of selenium are primarily mediated through selenoproteins [22], which exhibit antioxidant, anti-inflammatory, and immunoregulatory properties. Selenium is of great importance to human health [23] and for modulating immune responses [24,25,26]. Supplementation of Se has been found to promote the differentiation of CD4^+^ T cells into Th1 subsets [27]. Additionally, Se supplementation enhances the expression of glutathione peroxidase 4 (GPX4) in CD4^+^ T cells, increases the population of T follicular helper (TFH) cells, and further promotes immune responses following influenza vaccination in mice [28]. Additionally, selenium protects immune cells from oxidative stress and is beneficial for the survival of T cells and NK cells within highly oxidative environments, such as the tumor microenvironment [29].

L-selenomethionine (L-SeMet), an isomer of selenomethionine, is the primary form of selenium intake in humans. Since inorganic selenium compounds are inefficiently absorbed by the human body, selenomethionine, as an organic form of selenium, offers superior bioavailability. Research on selenomethionine has primarily focused on its antioxidant functions, emphasizing the role of certain selenoproteins in neutralizing reactive oxygen species (ROS) or regulating redox balance in immune cells [30]. Notably, dietary supplementation with selenomethionine has been demonstrated to markedly inhibit both the onset and spread of breast cancer [31].

Here, we report that the gut microbiota-derived metabolite L-selenomethionine (L-SeMet) can exert anti-tumor effects by enhancing the effector function of tumor-infiltrating CD8^+^ T cells. Specifically, by screening a collection of gut microbiota-derived metabolites, we identified L-SeMet as a positive modulator of CD8^+^ T cells. This occurred through the enhancement of LCK phosphorylation in CD8^+^ T cells. Treatment with L-SeMet in MC38 tumor-bearing mice resulted in significant tumor suppression. These data show that the gut microbiota-derived metabolite L-SeMet has the potential to improve the effects of cancer therapy.

## 2. Results

### 2.1. Metabolite Screen Identified L-SeMet as a Potent Activator of CD8^+^ T Cells

**Figure 1:**
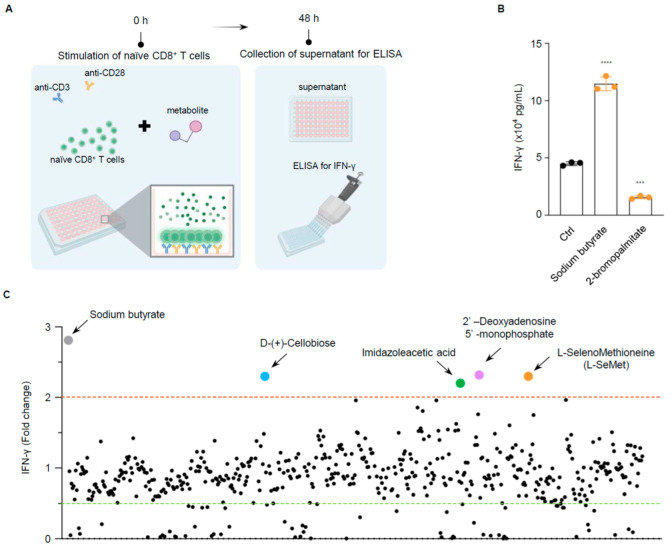

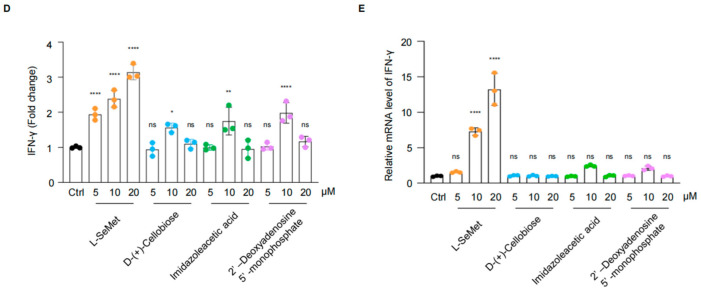
The metabolite screen identified L-SeMet as a potent activator of CD8^+^ T cells. (**A**) Schematic screening procedure: Splenic CD8^+^ T cells from C57BL/6 mice were treated with 544 bacteria-derived metabolites or a solvent control for 48 h. IFN-γ in the supernatants was detected using an ELISA assay. (**B**) Using the reported metabolites in our system, we detected IFN-γ secretion using an ELISA assay. Sodium butyrate, The reported positive regulator of CD8^+^ T cells, and 2-bromopalmitate, the reported negative regulator of CD8^+^ T cells. (**C**) Scatterplots displaying fold change in the secretion of IFN-γ across the collection of metabolites. Sodium butyrate acts as the positive control. Red line: The cutoff is a twofold activation relative to control. Green line: The cutoff is a 50% activation relative to control. (**D**) Scatterplots displaying fold change in the secretion of IFN-γ of the metabolites from screening. The concentrations of the metabolites are 5, 10, and 20 μM. (**E**) RT-qPCR analysis of *Ifn-γ* mRNA expression in CD8^+^ T cells treated with different metabolites. All scatterplots are shown as mean ± s.d.; n = three independent wells per experiment. Statistical differences were determined by one-way ANOVA; * *p* < 0.05; ** *p* < 0.01; *** *p* < 0.001; **** *p* < 0.0001; ns, no significant difference.

We screened a collection of metabolites derived from the gut microbiota to identify potential agonists of CD8^+^ T cells. This metabolite library included 554 compounds derived from enteric microorganisms (Table S1). To evaluate the impact of these metabolites on T cell function, we developed an in vitro screening system (Figure 1A). Naïve CD8^+^ T cells were isolated from mouse spleens and cultured in plates pre-coated with anti-CD3 and anti-CD28 antibodies in the presence of metabolites. The purity of the naïve CD8^+^ T cells had to exceed 95% (Figure S1A,B). Activated T cells rapidly produce inflammatory cytokines upon transitioning from their naïve state. As interferon γ (IFN-γ) is a key cytokine predominantly produced by cytotoxic T lymphocytes (CTLs), we measured IFN-γ levels using an ELISA assay as an indicator of CD8^+^ T cell effector function. To validate the system, we chose sodium butyrate [32], a microbiota metabolite reported to enhance CD8^+^ T cell function, and 2-Bromopalmitate (2-BP) [33], a metabolite known to inhibit CD8^+^ T cell activation. Consistent with previous studies, butyrate markedly amplified IFN-γ secretion by CD8^+^ T cells, whereas 2-BP had the opposite effect (Figure 1B). This indicates that the system we established is suitable for screening potential CD8^+^ T cell agonists.

The screening concentration was set to one-thousandth of the stock solution. Specifically, metabolites from plates 1 to 9 were screened at a concentration of 10 μM, plates 10 and 11 at 2 μM, and plates 12 and 13 at 3 μg/mL. This screening concentration was selected to ensure the nontoxicity of DMSO, the primary solvent, which was maintained at less than one-thousandth of the culture medium. Among the 554 gut microbiota metabolites, we identified four compounds that increased IFN-γ secretion in CD8^+^ T cells by more than twofold (Figure 1C). Further validation of these compounds revealed that L-selenomethionine (L-SeMet) exhibited the most pronounced enhancement of IFN-γ secretion at both the transcriptional and translational levels in a dose-dependent manner. Nevertheless, the other three compounds did not show a dose-dependent promotional effect (Figure 1D,E).

### 2.2. L-SeMet Promotes CD8^+^ T Cell Activation and Proliferation In Vitro

**Figure 2:**
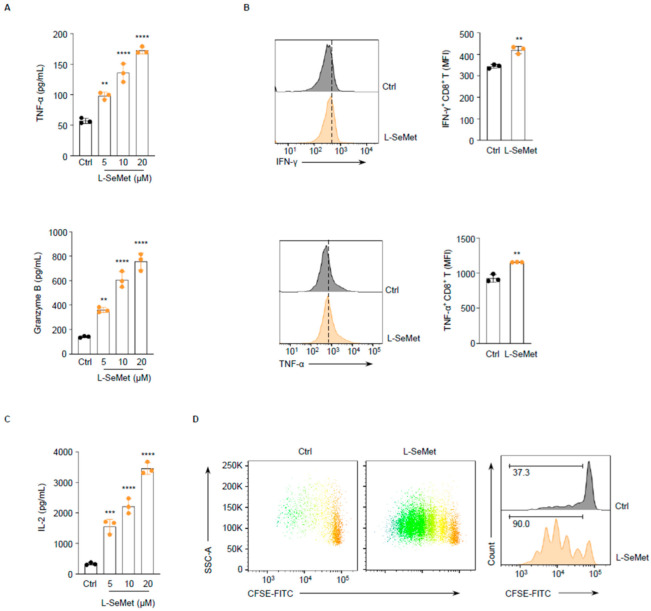
L-SeMet promotes T cell activation and proliferation in vitro. (**A**) Naïve CD8^+^ T cells from mice were activated by anti-CD3 and -CD28 antibodies in medium with or without L-SeMet for 48h and analyzed for the expression of the cytokines TNF-α (**top**) and GzmB (**bottom**) in culture supernatants using an ELISA assay. (**B**) Naïve CD8^+^ T cells from mice were activated with or without L-SeMet, then treated with BFA and analyzed for the expression of the cytokines IFN-γ (**top**) and TNF-α (**bottom**) using FACS. The dashed line represents the fluorescence intensity of L-SeMet treated group. (**C**) Naïve CD8^+^ T cells were activated for 48 h and analyzed for the expression of IL-2 in supernatants using an ELISA assay. (**D**) Naïve CD8^+^ T cells were activated in medium with or without L-SeMet for 72 h. Cell proliferation (CFSE) was determined by CFSE staining. Data are shown as mean ± s.d.; n = three independent wells per experiment. Statistical differences were determined by one-way ANOVA (**A**,**C**) and unpaired two-tailed Student’s *t*-test (**B**); ** *p* < 0.01; *** *p* < 0.001; **** *p* < 0.0001.

We further validated the ability of L-SeMet to enhance CD8^+^ T cell function in vitro. First, we assessed whether L-SeMet could promote the secretion of additional key cytokines by CD8^+^ T cells. Consistent with our screening results, L-SeMet enhanced the secretion of tumor necrosis factor α (TNF-α) and granzyme B (GzmB) in a dose-dependent manner (Figure 2A). To evaluate intracellular cytokine accumulation, Brefeldin A (BFA) was added after naïve T cell activation to block the secretion of effector molecules. We observed that intracellular IFN-γ and TNF-α were also increased in the L-SeMet-treated group (Figure 2B). These findings indicate that L-SeMet effectively promotes the activation of CD8^+^ T cells.

Activated T cells must rapidly proliferate to fulfill their functional roles. Interleukin 2 (IL-2) is a key growth factor for CD8^+^ T cells. We found that IL-2 secretion by CD8^+^ T cells co-incubated with L-SeMet increased in a concentration-dependent manner (Figure 2C). Additionally, CFSE labeling of T cells revealed that pre-treated with L-SeMet enhanced the T cell proliferation rate (Figure 2D). Collectively, these results indicate that L-SeMet enhances both the activation and proliferation of CD8^+^ T cells in vitro.

### 2.3. L-SeMet Potentiates the Cytotoxicity of CD8^+^ T Cells

**Figure 3:**
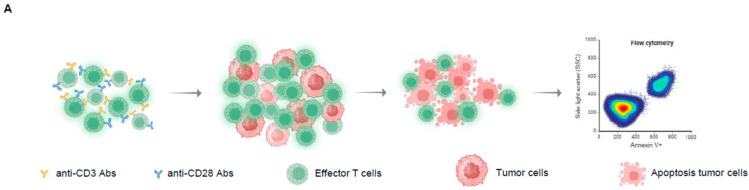

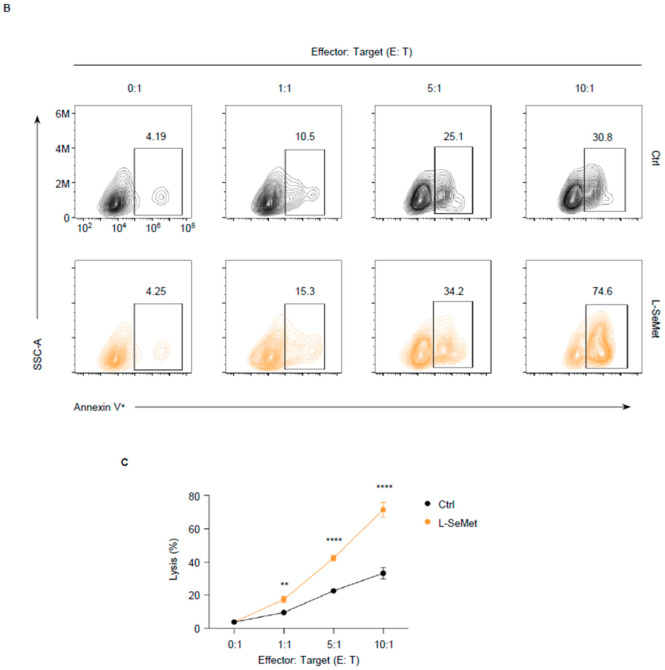
L-SeMet potentiates the cytotoxicity of CD8^+^ T cells. (**A**) Experimental scheme of the killing effect of mouse cytotoxic T cells on target tumor cells in vitro. Naïve CD8^+^ T cells from mice were activated with or without L-SeMet and then co-cultured with pre-loaded MC38 cells. The apoptosis of MC38 cells was detected using the Annexin V assay. (**B**,**C**) In vitro cytotoxicity of CD8^+^ T cells (effector) cultured with MC38 cells (target). The percentage of annexin V^+^ MC38 cells was measured by FACS analysis. Representative flow cytometry plots are shown of different effector-to-target ratios (E:T), 0:1, 1:1, 5:1, and 10:1 (**B**). The percentages of cells in the boxed areas are indicated (**C**). All data are the mean ± s.d.; n = three independent wells per experiment. Two-way ANOVA; ** *p* < 0.01; **** *p* < 0.0001.

CD8^+^ T cells play a vital role in antitumor immunity by specifically recognizing tumor antigens and killing tumor cells. A critical indicator of CD8^+^ T cell effector function is their ability to lyse tumor cells. To determine whether L-SeMet enhanced the cytotoxic activity of CD8^+^ T cells, we established an in vitro co-culture system of CD8^+^ T cells and tumor cells (Figure 3A). Activated CD8^+^ T cells were pretreated with L-SeMet and then co-incubated with MC38 tumor cells, after which the apoptosis of MC38 cells was assessed. Annexin V, which specifically binds to phosphatidylserine (PS) on apoptotic cells, was used as a marker for apoptosis. The results showed that as the effector-to-target (E:T) ratio increased, the apoptosis of the target MC38 cells intensified. At the same E:T ratio, MC38 cells co-incubated with L-SeMet-pretreated CD8^+^ T cells showed notably higher levels of apoptosis (Figure 3B,C). The concentration of L-SeMet utilized did not exhibit toxicity toward CD8^+^ T cells (Figure S2B). Additionally, to test whether L-SeMet exerts a direct cytotoxic effect on MC38 cells, we co-cultured L-SeMet and MC38 cells for different concentrations and times and assessed the survival of MC38 cells using Annexin V and CCK8. Our results showed that L-SeMet at different concentrations had no cytotoxic effect on MC38 cells (Figure S2C–E). The representative flow cytometry gating strategy to identify apoptotic MC38 cells is shown (Figure S2A). These findings indicate that L-SeMet significantly enhances the tumor-killing ability of CD8^+^ T cells in vitro.

### 2.4. LCK Signaling Mediates L-SeMet-Promoted CD8^+^ T Cell Activation and Responses

**Figure 4:**
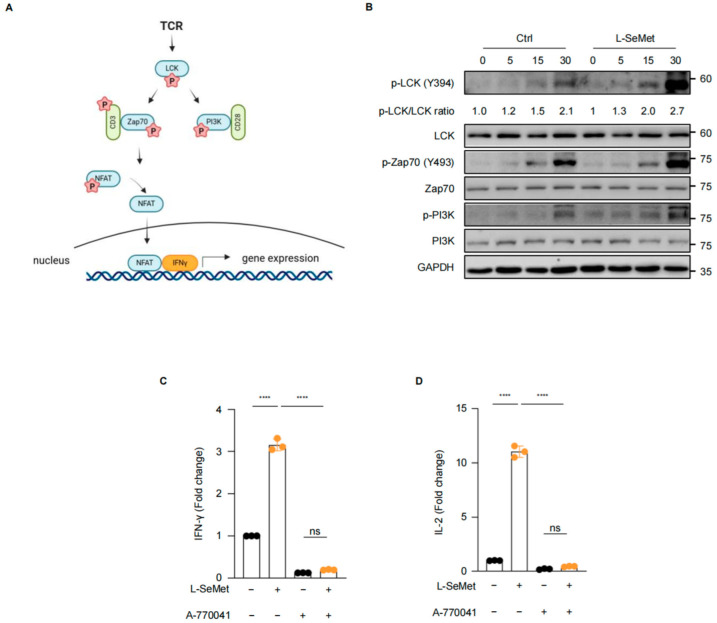
LCK signaling mediates L-SeMet-promoted CD8^+^ T-cell activation and responses. (**A**) TCR signaling that initiates T cell activation. P represents phosphorylation. (**B**) Naïve CD8^+^ T cells were stimulated (2 μg/mL anti-CD3 and 1 μg/mL anti-CD28 antibodies) with or without L-SeMet for the indicated times, and protein expression was analyzed by Western blotting. (**C**,**D**) Naïve CD8^+^ T cells treated with L-SeMet and/or A-770041. The secretion of IFN-γ (**C**) and IL-2 (**D**) was detected by ELISA. All data are the mean ± s.d.; n = three independent wells per experiment. One-way ANOVA; **** *p* < 0.0001; ns, no significant difference.

T cell activation is initiated when antigen-presenting cells (APCs) process and present antigens as peptide-MHC complexes to T cells. Briefly, these complexes bind to the T cell receptor (TCR) on T cells, triggering downstream signaling. Upon TCR engagement, the first molecule recruited to the TCR-CD3 complex is leukocyte C-terminal Src kinase (LCK), a member of the SRC family kinases (SFK). Subsequently, LCK undergoes phosphorylation, and recruits and phosphorylates the zeta chain of T cell receptor-associated protein kinase 70 (ZAP70) and the phosphatidylinositol 3-kinase pathway (PI3K). Phosphorylated ZAP70 further activates a cascade of downstream protein complexes, culminating in T cell activation [34]. The activation of ZAP70 leads to the dephosphorylation of nuclear factors of activated T cells (NFAT). NFAT then translocates into the nucleus to initiate IFN-γ gene expression [35,36] (Figure 4A).

We hypothesized that L-SeMet enhances the effector functions of CD8^+^ T cells by strengthening the activation of the TCR signaling pathway. Therefore, we analyzed the TCR signaling pathway in L-SeMet-treated CD8^+^ T cells. Naïve CD8^+^ T cells sorted from mouse spleens were stimulated with anti-CD3/28 antibodies for 5, 15, and 30 min. Cells collected at each time point were subjected to Western blot analysis to detect the phosphorylation of key proteins in the TCR signaling pathway. Our results showed that in naïve CD8^+^ T cells from the control group, the phosphorylation levels of key TCR signaling proteins, including LCK (Y394), ZAP70, and PI3K, gradually increased with anti-CD3/28 activation. Remarkably, co-stimulation of CD8^+^ T cells with L-SeMet led to a significant enhancement in the phosphorylation levels of LCK (Y394), ZAP70, and PI3K during activation (Figure 4B). Additionally, we added the LCK inhibitor A-770041 [37] to L-SeMet-treated CD8^+^ T cells and found that the addition of the LCK inhibitor completely abolished T cell activation promoted by L-SeMet (Figure 4C,D). Taken together, these results suggest that L-SeMet amplifies TCR signaling in CD8^+^ T cells, indicating that L-SeMet enhances T cell activation via LCK-mediated pathways.

### 2.5. L-SeMet Inhibits Colorectal Cancer Growth by Enhancing CD8^+^ T Cell Effector Functions

**Figure 5:**
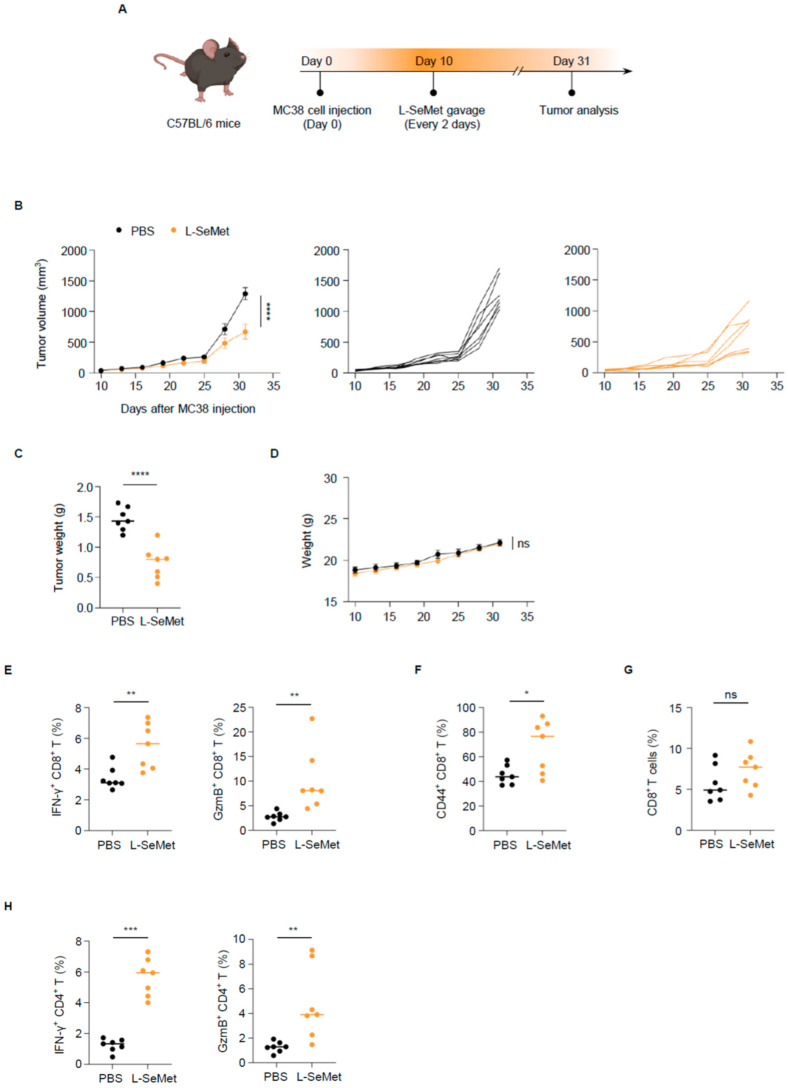
L-SeMet inhibits colorectal cancer growth by enhancing CD8^+^ T cell effector functions. (**A**) Schematic experimental procedure in (**B**–**H**): Mice were inoculated with MC38 cells at day 0 and were gavage with L-SeMet or PBS every 2 days. At day 31, the tumor was harvested. (**B**) Tumor growth curves: Effect of L-SeMet administration compared to that of the control group on MC38 tumor growth in C57BL/6 mice (left), and the tumor growth curves of every mouse treated with PBS (middle) and L-SeMet (right). (**C**) Tumor weight. At day 31, after inoculation of MC38 cells, the tumors were harvested and weighed. (**D**) Mice weight. During administration, the weight of the mice was recorded. (**E**–**H**) Tumor-infiltrating immune cells were examined: Percentage of IFN-γ^+^, GzmB^+^ cells among CD8^+^ T cells (**E**), CD44^+^ cells among CD8^+^ T cells (**F**), and CD8^+^ T cells among CD45^+^ T cells (**G**). Percentage of IFN-γ^+^ and GzmB^+^ cells among CD4^+^ T cells (**H**); n = 7. Each symbol in (**E**–**H**) represents a measurement from an individual mouse. Data in (**B**,**D**) are the mean ± s.e.m.; data in (**C**,**E**–**H**) are the mean ± s.d. Statistics were analyzed by two-way ANOVA (**B**,**D**) and unpaired two-tailed Student’s *t*-test (**C**,**E**–**H**); * *p* < 0.05; ** *p* < 0.01; *** *p* < 0.001; **** *p* < 0.0001; ns, no significant difference.

Given that L-SeMet enhances the effector functions and tumor-killing ability of CD8^+^ T cells in vitro, we sought to investigate whether L-SeMet could suppress tumor growth in vivo. The C57BL/6 mice were subcutaneously inoculated with MC38 cells at day 0 and then treated with L-SeMet via oral gavage every other day at day 10 (Figure 5A). Importantly, tumor growth was significantly suppressed by L-SeMet compared to that in the control group (Figure 5B). No obvious difference in body weight was observed between the treated and control mice (Figure 5D), indicating that the drug is non-toxic to mice. At day 31, after the inoculation of MC38 cells, we sacrificed the mice and isolated the tumors. Tumor weights in the L-SeMet treatment group were lower than those in the control group (Figure 5C). We then analyzed the immune cell infiltration in the tumor microenvironment (TME) and found that the percentage of tumor-infiltrating effector CD8^+^ T cells, which express IFN-γ, GzmB, and CD44, was elevated in L-SeMet-treated mice (Figure 5E,F). However, the overall proportion of CD8^+^ T cells among CD45^+^ cells remained unchanged (Figure 5G). These results indicate that L-SeMet suppresses colorectal tumor growth probably by enhancing the effector functions of CD8^+^ T cells within the TME rather than by promoting their infiltration. Furthermore, L-SeMet treatment enhanced the ability of CD4^+^ T cells to secrete IFN-γ and GzmB (Figure 5H). L-SeMet had no obvious effect on CD4^+^ T cells in vitro (Figure S1C). Therefore, the enhanced cytokine secretion ability of tumor-infiltrating CD4^+^ T cells in the treatment group may not be directly caused by L-SeMet but rather indirectly influenced by CD8^+^ T cells. Additionally, no significant changes were observed in NK cells, NKT cells, macrophages, or MDSCs (Figure S3C). The representative flow cytometry gating strategies used to identify tumor-infiltrating immune cells are shown (Figure S3A,B). These findings suggest that L-SeMet promotes the secretion of effector molecules by T cells while having no notable effect on the infiltration of other immune cells. Collectively, these findings support that L-SeMet inhibits colorectal cancer development by improving the anti-tumor immune responses of CD8^+^ T cells.

## 3. Discussion

L-Selenomethionine (L-SeMet) has been shown to modulate immune responses by influencing CD4^+^ T cells and NK cell activity. Here, we demonstrated the direct effect of L-SeMet in enhancing CD8^+^ T cell effector functions and its efficacy in suppressing MC38 tumor growth, expanding our understanding of multifaceted impact of L-SeMet among different immune cell types.

In this study, we initially screened a repository of gut microbial metabolites and identified L-SeMet as a metabolite that significantly enhances CD8^+^ T cell effector functions. We showed that L-SeMet exerts its effects during the early stages of T cell activation by promoting TCR signaling in CD8^+^ T cells. Furthermore, we confirmed that L-SeMet effectively suppresses the growth of MC38 tumors, highlighting its potential as a candidate for anti-tumor drug development.

Most known biological activities of SeMet are linked to its incorporation into selenoproteins. Several selenoproteins function as antioxidants, protecting cells against oxidative stress. For instance, glutathione peroxidase (GPX), a selenoprotein, inhibits ROS production in T cells, and its deficiency leads to impaired TCR activation in T cells [30]. Moreover, dietary selenium supplementation has been shown to upregulate translation factor genes for selenoprotein K (SELK) in human peripheral blood leukocytes, suggesting a role for SELK in enhancing lymphocyte function [38]. Therefore, we hypothesize that the effects of SeMet on CD8^+^ T cells may also result from its role in the synthesis of selenoproteins that regulate CD8^+^ T cell function.

Numerous gut microbes utilize selenium, converting it into selenomethionine (SeMet), selenocysteine (SeCys), and other seleno-amino acids and selenoproteins. Dietary selenium supplementation alters gut microbiota composition, which in turn influences host selenium levels [39]. *Saccharomyces cerevisiae* can convert inorganic selenium into selenomethionine, and oral supplementation with selenium-enriched yeast reduces intestinal inflammation [40] and slows the progression of colorectal cancer [41] in mice. In our study, L-SeMet effectively suppressed MC38 tumor growth, suggesting that exploring the role of SeMet-producing microorganisms in MC38 tumor inhibition warrants further investigation.

The role of L-SeMet in tumor killing remains highly controversial. Some studies have shown that selenite can induce apoptosis in tumor cells [42,43,44]. However, other studies have shown that selenium may support tumor cell survival. Culturing triple-negative breast cancer cells in selenium-containing media enhances their colony-forming ability [45]. Additionally, selenium concentrations in tumor tissues are nearly four times higher than in normal cells [46]. Therefore, it is still unclear whether selenium compounds exert direct cytotoxic effects on tumor cells. Our findings indicate that co-incubation of L-SeMet with MC38 tumor cells for 24 h in vitro did not result in significant cytotoxicity against the tumor cells. To further elucidate whether L-SeMet suppresses MC38 tumor growth by enhancing the function of tumor-infiltrating CD8^+^ T cells, additional experiments are required. Specifically, we need to use immunodeficient mice or block CD8^+^ T cells with CD8a antibodies and subsequently evaluate the effect of L-SeMet on tumor growth to validate this mechanism.

## 4. Materials and Methods

### 4.1. Experimental Animals

All mice used in this research were derived from a C57BL/6 genetic background. Wild-type C57BL/6 mice were obtained from Beijing Vital River Laboratory Animal Technology. Animals were housed under specific-pathogen-free conditions, with strictly controlled temperature (21–23 °C), humidity (30–60%), and light cycle (12-h light/dark). For all experiments, only mice aged 8 to 12 weeks were selected to ensure uniformity. All procedures involving animals were conducted following approval from the Institutional Animal Care and Use Committee of Nanhu Laboratory.

### 4.2. In Vitro T Cell Isolation

Spleens from wild-type C57BL/6 mice were first homogenized, and the resulting tissue was filtered through a 40-μm cell strainer to generate a single-cell suspension. To remove red blood cells, ACK lysis buffer was used. The splenocytes were processed using the naïve CD8a^+^ T cell Isolation kit (Miltenyi, Bergisch Gladbach, Germany) to obtain purified mouse naïve CD8^+^ T cells and the CD4^+^ T cell Isolation kit for CD4^+^ T cells. The purity of the isolated cells was >95%.

### 4.3. T Cell Culture and Function Analysis

Purified anti-CD3 and anti-CD28 monoclonal antibodies (mAbs) (BioLegend, San Diego, CA, USA) were used to activate mouse naïve CD8^+^ T cells from mice in vitro. The antibodies were applied to a 96-well plate, with final concentrations of 2 μg/mL for anti-CD3 and 1 μg/mL for anti-CD28, and incubated overnight at 4 °C. The purified mouse naïve CD8^+^ T cells were plated at a density of 1 × 10^5^ cells per well into the pre-coated plate and cultured for 48 h at 37 °C in the presence of metabolites (MCE). The same protocols for activation and culture conditions were used for CD4^+^ T cells. The supernatant was then collected for the analysis of IFN-γ, TNF-α, GzmB, and IL-2 levels using ELISA according to the manufacturer’s protocol (BioLegend). Additionally, cells were harvested for flow cytometry analysis or qPCR to detect cytokine expression.

### 4.4. Flow Cytometry

For in vitro stimulation assays, the purified CD8^+^ T cells were activated with anti-CD3/28 mAbs in the presence of metabolites then the BFA/monensin mixture was added. After stimulation, the cells were incubated with fluorescently labeled antibodies targeting surface molecules for 30 min at 4 °C. For intracellular staining, after surface marker staining, cells were fixed and permeabilized using a Fixation/Permeabilization kit (BD, Franklin Lakes, NJ, USA), followed by staining for intracellular proteins.

For tumor-infiltrating lymphocyte analysis, the tumor was minced and digested into a single-cell suspension using a Tumor Dissociation Kit (Miltenyi). The cell suspension was filtered through a 70-μm cell strainer. The sorted cells were treated with anti-CD16/32 mAbs (BD) to block Fc receptors, and then stained with fluorescent antibodies against surface antigens for 30 min at 4 °C. Viability dye was utilized to exclude dead cells. For intracellular cytokine analysis, cells were fixed and permeabilized according to the manufacturer’s instructions using the Fixation/Permeabilization kit (BD), and stained for intracellular molecules. The following antibodies obtained from BioLegend were used for flow cytometry: Alexa Fluor 700 anti-mouse CD45, Brilliant violet 421 anti-mouse CD4, Brilliant violet 605 anti-mouse CD8, APC anti-mouse IFN-γ, PE anti-mouse TNF-α, FITC anti-mouse Granzyme B, PerCP anti-mouse CD44, PE anti-mouse CD3, Brilliant violet 605 anti-mouse CD11b, APC anti-mouse LY6G, Brilliant violet 421 anti-mouse LY6C, FITC anti-mouse NK1.1, and PerCP-cy5.5 anti-mouse F4/80. All data were analyzed with Flowjo (v10.8.1, BD, Franklin Lakes, NJ, USA) software.

### 4.5. CD8^+^ T-Cell Cytotoxicity Assay

Naïve CD8^+^ T cells were activated with or without L-SeMet (20 μM) for 48 h. Then, the activated CD8^+^ T cells were collected and washed using fresh medium. Then, the activated CD8^+^ T cells were co-cultured with MC38 cells for 18 h. The cytotoxic efficiency of CD8^+^ T cells was measured using an Annexin V assay by flow cytometry.

### 4.6. Proliferation Analysis by CFSE Assay

Isolated CD8^+^ T cells were incubated with a 5 μM CFSE solution, and the staining reaction was terminated by adding five volumes of pre-warmed culture medium (with 2% FBS). Following incubation, the cells were transferred to a 96-well plate pre-coated with anti-CD3/28 mAbs. After 72 h, the cells were analyzed using flow cytometry. The CFSE staining was carried out following the protocol provided by the manufacturer (Invitrogen, Carlsbad, CA, USA).

### 4.7. Tumor Growth and Treatments

A total of 5 × 10^5^ MC38 cells were implanted subcutaneously into the right flank of mice. Tumor dimensions were recorded using a vernier caliper, with length (a) and width (b) measured to calculate tumor volume using the formula: tumor volume = ab^2^/2. Once the tumor reached approximately 100 mm^3^, mice were gavage with L-SeMet (10 mg/kg body weight) every other day. All tumor measurements and animal procedures adhered to ethical guidelines, and mice were euthanized while tumor sizes remained below 1500 mm^3^. The study was conducted with approval from the Institutional Animal Care and Use Committee of Nanhu Laboratory.

### 4.8. Western Blot

Naïve CD8^+^ T cells were sorted and stimulated with anti-CD3 and anti-CD28 mAbs at concentrations of 2 μg/mL and 1 μg/mL for 5 min, 15 min, and 30 min, with or without L-SeMet at 37 °C. The cells were then harvested, followed by lysis using RIPA for 20 min on ice. The samples were analyzed by western blotting.

### 4.9. Statistical Analysis

GraphPad Prism 9.0 (GraphPad, San Diego, CA, USA) was used for data analysis and image processing. The statistical tests and *p* values are denoted in each figure legend: * *p* <0.05; ** *p* < 0.01; *** *p* <0.001, and ns for no significance.

## Data Availability

Data will be made available on request.

## Supplementary Materials

The following supporting information can be downloaded at: https://www.mdpi.com/article/10.3390/ijms26062511/s1.
