# Supplementary material for: Microbiota-Derived L-SeMet Potentiates CD8+ T Cell Effector Functions and Facilitates Anti-Tumor Responses
Source: Int J Mol Sci. 2025 Mar 11;26(6):2511. doi: 10.3390/ijms26062511 (PMC11941941; doi:10.3390/ijms26062511)
Supplement: Supplementary file 1 [file ijms-26-02511-s001.zip › Figure legends.pdf]

Fig. S1 Isolation and functional analysis of T cells. **A** Gating strategy to identify the proportion of $CD8^+$ T cells in all spleen cells. **B** Gating strategy to identify the purity of $CD8^+$ T cells after isolating from the spleen using naïve $CD8a^+$ T cell isolation kit (Miltenyi). **C** Naïve $CD4^+$ T cells were activated for 48h, and analyzed the expression of the IL-2 in supernatants using ELISA assay. Data are shown as mean±s.d.; n=3 independent wells per experiment. Statistical differences were determined by one-way ANOVA (**C**); ns, no significant difference.

Fig. S2 Effect of L-SeMet on the tumor-killing activity of $CD8^+$ T cells in vitro. **A** The representative flow cytometry gating strategies to identify the apoptosis MC38 cells in vitro. **B-C** The percentage of annexin V^+ $CD8^+$ T cells (**B**) and MC38 cells (**C**) treated with L-SeMet were measured by FACS analysis. Representative flow cytometry plots (**Left**) and percentages of cells in boxed areas (**Right**) are shown. **D-E** The OD value of CCK8. MC38 cells treated with L-SeMet in different concentration for 24 h (**D**) and 72 h (**E**), then add CCK8 (MCE) into the culture medium. Detect the OD value at 450 nm. Data are shown as mean±s.d.; n=3 independent wells per experiment. Statistical differences were determined by One-way ANOVA (**D**) and unpaired two-tailed Student's *t*-test (**B, C, E**); ns, no significant difference.

Fig. S3 Anti-tumor effect of L-SeMet in vivo. **A-B** The representative flow cytometry gating strategies to identify tumor-infiltrating immune cells. **C** Tumor-infiltrating immune cells were examined. The percentage of NK cells, NKT cells, M-MDSC, PMN-MDSC, macrophage among $CD45^+$ T cells. n=7. Each symbol represents measurement from an individual mouse. All data are the mean±s.d.. Statistics are analyzed by unpaired two-tailed Student's *t*-test; ns, no significant difference.
