# Supplementary material for: Microbiota-Derived L-SeMet Potentiates CD8+ T Cell Effector Functions and Facilitates Anti-Tumor Responses
Source: Int J Mol Sci. 2025 Mar 11;26(6):2511. doi: 10.3390/ijms26062511 (PMC11941941; doi:10.3390/ijms26062511)
Supplement: Supplementary file 1 [file ijms-26-02511-s001.zip › Supplementary figures.pdf]

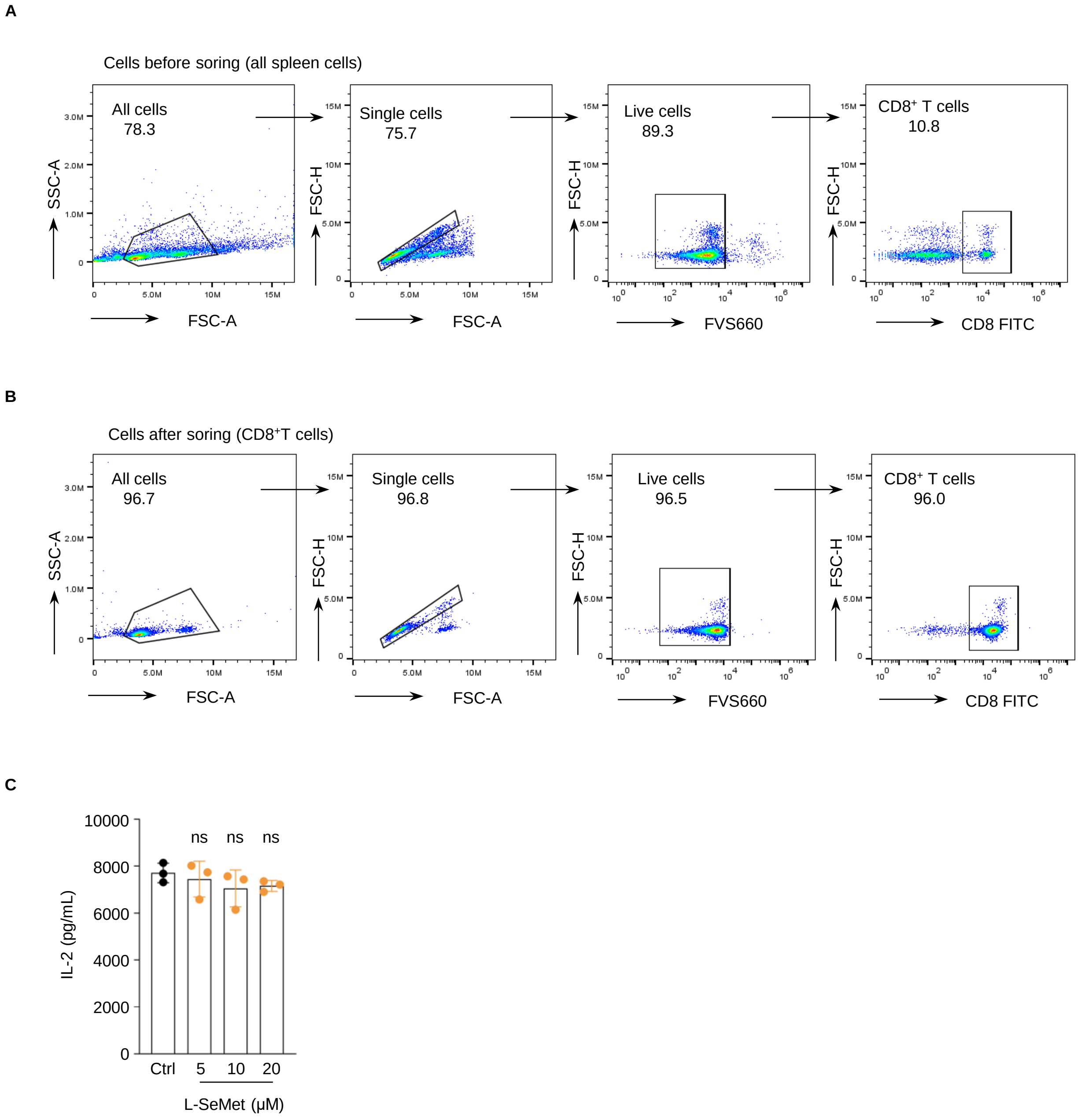

Figure. S1 Isolation and functional analysis of T cells

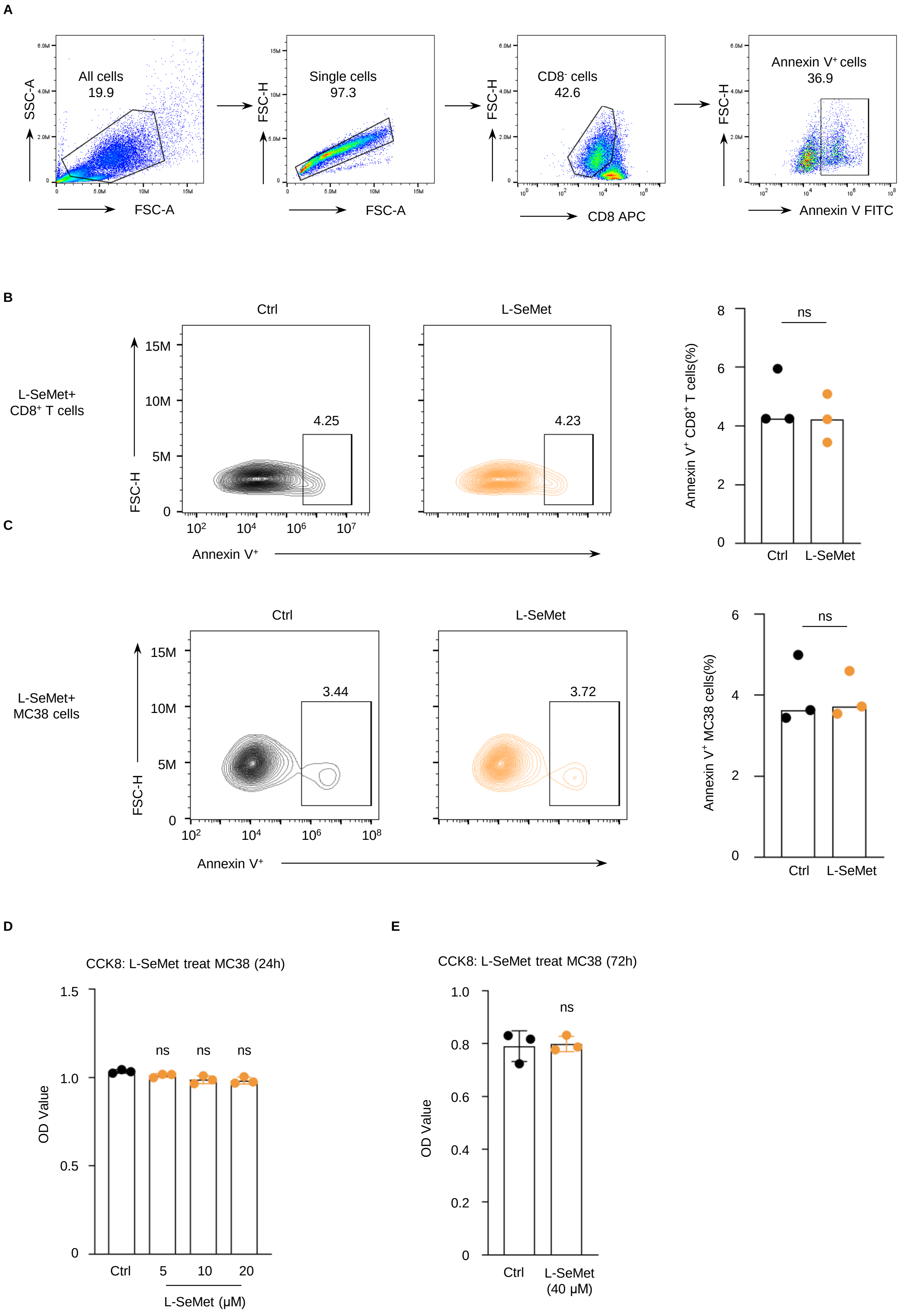

Figure. S2 Effect of L-SeMet on the tumor-killing activity of CD8$^+$ T cells in vitro

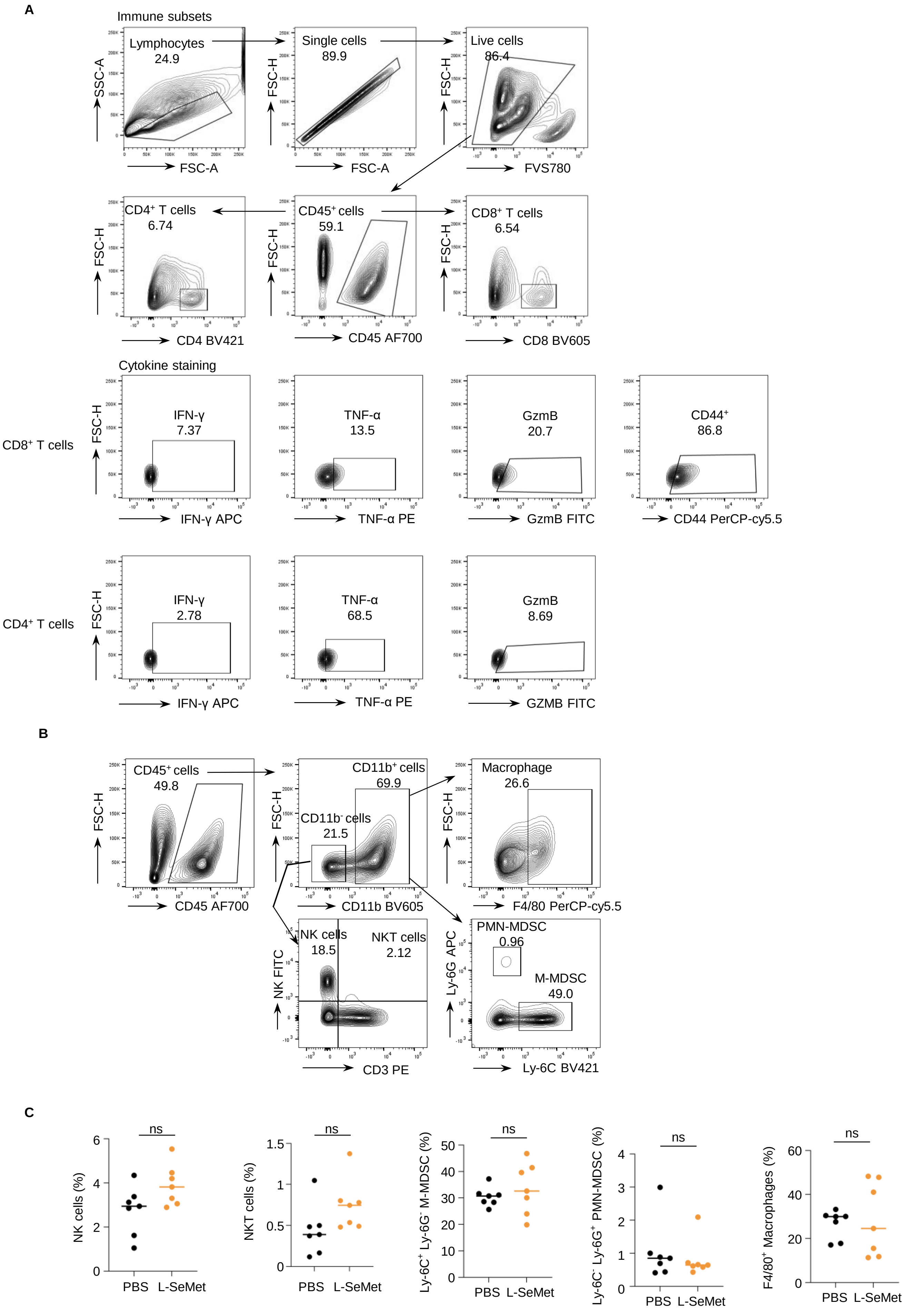

Figure. S3 Anti-tumor effect of L-SeMet in vivo
